# Expandable self-locking nail in the management of closed diaphyseal fractures of femur and tibia

**DOI:** 10.4103/0019-5413.53457

**Published:** 2009

**Authors:** Sudhir K Kapoor, Himanshu Kataria, Tankeswar Boruah, Satya R Patra, Aashish Chaudhry, Saurabh Kapoor

**Affiliations:** Department of Orthopaedics, Lady Hardinge Medical College and Dr Ram Manohar Lohia Hospital, Trauma Care Centre & PGIMER, New Delhi, India

**Keywords:** Diaphyseal fracture femur, diaphysial fracture tibia, expandable nail, self-locking nail, radiation risk

## Abstract

**Background:**

Intramedullary fixation is the treatment of choice for closed diaphyseal fractures of femur and tibia. The axial and rotational stability of conventional interlocking nails depends primarily on locking screws. This method uses increased operating time and increased radiation exposure. An intramedullary implant that can minimize these disadvantages is obviously better. Expandable intramedullary nail does not rely on interlocking screws and achieves axial and rotational stability on hydraulic expansion of the nail. We analyzed 32 simple fractures of shaft of femur and tibia treated by self-locking expandable nail.

**Materials and Methods:**

Intramedullary fixation was done by using self-locking, expandable nail in 32 patients of closed diaphyseal fractures of tibia (n = 10) and femur (n = 22). The various modes of injury were road traffic accidents (n = 21), fall from height (n = 8), simple fall (n = 2), and pathological fracture (n = 1). Among femoral diaphyseal fractures 16 were males and six females, average age being 33 yrs (range, 18- 62 yrs). Seventeen patients had AO type A (A1 (n = 3), A2 (n = 4), A3 (n = 10)) and 5 patients had AO type B (B1 (n = 2), B2 (n = 2), B3 (n = 1)) fractures. Eight patients having tibial diaphyseal fractures were males and two were females; average age was 29.2 (range, 18- 55 yrs). Seven were AO type A (A1 (n = 2), A2 (n = 3), A3 (n = 2)) and three were AO type B (B1 (n = 1), B2 (n = 1), and B3 (n = 1)). We performed closed (n = 27) or open reduction (n = 5) and internal fixation with expandable nail to stabilize these fractures. The total radiation exposure during surgery was less as no locking screws were required. Early mobilisation and weight-bearing was started depending on fracture personality and evidences of healing. Absence of localised tenderness and pain on walking was considered clinical criteria for union, radiographic criteria of union being continuity in at least in three cortices in both AP and lateral views. Patients were followed for at least one year.

**Results:**

The average operative time was 90 min (range, 55-125 min) for femoral fractures and 53 min (range, 25-115 min) for tibial fractures. Radiation exposure was minimum, average being 84 seconds (range, 54-132) for femoral fractures and 54 seconds (range, 36-78) for tibial fractures. All fractures healed, but few had complications, such as infection (one case with tibial fracture) bent femoral nail with malunion (n = 1), and delayed union (n = 3; 2 cases in femur and 1 case in tibia). Mean time of union was 5.1 months (range, 4-10½ months) for femoral fractures and 4.8 months (range, 3-9 months) for tibial fractures.

**Conclusion:**

We found the nail very easy to use with effective fixation in AO type A and B fractures in our setting. Less surgical time is required with minimum complications. The main advantage of the expandable nail is that if affords. satisfactory axial, rotatory, and bending stability with decreased radiation exposure to operating staff and the patient.

## INTRODUCTION

**Figure 1:**
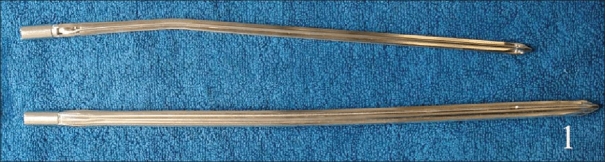
Photograph showing expandable nail for tibia with posterior bend in its proximal third (above) and for femur with anterior bowing (below).

Interlocked intramedullary nailing is the most commonly used fixation modality for the operative treatment of closed diaphyseal fractures of femur and tibia.^1^ It allows for early weight-bearing, quick rehabilitation, and good healing rate with minimum complications. It is ideally used by closed method without exposing the fracture site. However, the insertion of the nail and subsequent placement of locking screws at its two ends are associated with significant radiation exposure and a long operation time.^2^ An intramedullary device that can overcome these inherent disadvantages of interlocked intramedullary nail is obviously better. A hydraulically expandable nail is one such device.^3^ It consists of an expandable stainless steel tube with four reinforcement bars connected by thin folded stainless steel membranes, with a conical distal end.^4^ Normal saline is injected into the hollow inner core of the nail under pressure through a proximal valve to expand it and increase its effective diameter [Figure 1].

This stainless steel nail is introduced into the medullary canal in its collapsed form for easy insertion, with or without reaming. Once in proper position, it is gradually expanded by introducing pressurized saline with the help of a hand-operated pump. A pressure gauge, inbuilt in the pump, monitors the pressure. The nail can be expanded to approximately one and half times of its original diameter [Table 1]. Once expanded, it conforms to the configuration of the medullary canal, providing close fit along the entire length of the canal. The nail, after expansion, has mechanical characteristics of a large diameter nail and provides good torsional and rotational stability, along the entire length of the bone without the need for interlocking screws.^5^ The expandable nail thus does away with the need for fluoroscopy required in placement of interlocking screws. Consequently, this technique can be used as an alternative to conventional interlocking nails, with minimum radiation hazard to the surgeon, other operation room (OR) personnel, and the patient. This technology has been used with encouraging success in the treatment of femur, tibia, and humeral shaft fractures.^5^–^7^

**Table 1:** Recommended diameter of nails for both femur and tibia as per the size of the medullary canal

	Reduced diameter (mm)	Maximal inflated diameter (mm)	Recommended for isthmus diameter (mm)
Tibia*	8.5	13.5	10.5-12.0
	10.0	16.0	12.0-14.0
Femur†	8.5	13.5	10.5-12.0
	10.0	16.0	12.0-14.0
	12.0	19.0	14.0-17.0

* Tibial nails are angled 8.5° at the proximity of their proximal one third,

† The femoral nails have an anterior bowing of 1.5 m radius.

We present our experience of using this self-locking expandable nail in the treatment of 32 simple diaphyseal fractures of femur and tibia (classified as per AO classification).^8^ We analyzed the operative technique, amount of radiation exposure, and total operation time.

## MATERIALS AND METHODS

From April 2005 to December 2007, 32 closed diaphyseal fractures (AO type A and B) of femur (22) and tibia (10) were stabilized with this expandable nail.

**Figure 2:**
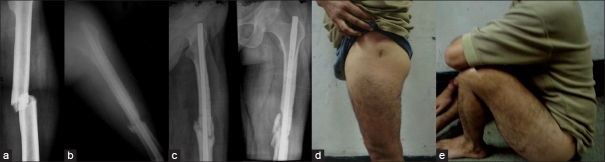
(a) Preoperative anteroposterior X-ray of thigh showing a transverse fracture soft femur. (b) Post-operative antero-posterior X-rays of thigh showing well aligned fracture with intramedullary expandable nail. (c) Anteroposterior and lateral view follow up X-rays showing fracture union. (d,e) clinical result at final follow up showing good functional outcome

**Figure 3:**
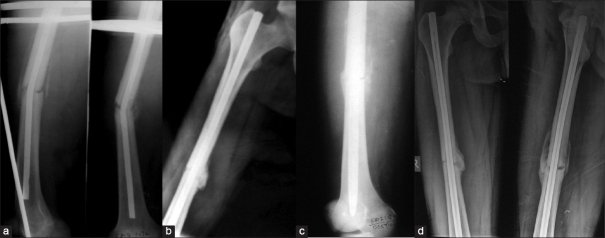
(a) Case of implant failure with bent K-nail; (b,c) postoperative radiographs after implant removal with well aligned fracture and expandable nail in place. (d) Follow up X-rays showing radiological union

Out of 22 diaphyseal femur fractures (two of them more than three weeks old), 16 were males and six were females. The average age of the patients was 33 years (range 18 — 62 years). Seventeen patients had type A (A1 (n = 3), A2 (n = 4), A3 (n = 10)) and 5 patients AO type B (B1 (n = 2), B2 (n = 2), B3 (n = 1)) fractures. Out of 22 cases, closed nailing was attempted in 20 cases and succeeded in 18 [Figure 2a–e]. In two patients open reduction had to be done. One had a long oblique fracture with soft tissue interposition while the other was a very obese patient in whom satisfactory closed reduction could not be achieved. In two patients of femur fractures, open reduction was planned from the beginning, one was a case of implant failure where the broken plate had to be removed prior to nailing and the other was a case which presented to us four and half weeks after the trauma. Cancellous bone graft, harvested from the iliac crest was used in both the cases. Among the closed nailing cases one patient presented with a bent K-nail and mal-uniting fracture. The nail was manually straightened by closed manipulation under anaesthesia at the beginning of the surgery. The K-nail was then removed over a guide wire, reaming was done over the same guide wire up to a maximum possible 10 mm diameter and subsequently a 8.5 mm expandable nail was put after removal of the guide wire [Figure 3a–d]. A 8.5 mm nail was chosen for this case as it can be expanded up to a maximum of 13.5 mm diameter [Table 1].

All closed nailing of the femur were performed on fracture table with the patient in supine position. The femoral nail was introduced through the tip of the greater trochanter in an antegrade manner. In the two cases where open reduction was pre-planned, surgery was done in lateral position on a radiolucent operating table. Here the fracture was opened through a posterolateral approach, although the nail was introduced in an antegrade manner as in other cases.

Ten patients of tibial diaphyseal fractures were treated with expandable nail which differs from the nail used in femur in having a posterior bend in proximal one-third. Eight patients were male and two were females. The average age of the patient's was 29.2 years (range, 18-55 years). Seven were AO type A (A1 (n = 2), A2 (n = 3), A3 (n = 3)) and three were B (B1 (n = 1), B2 (n = 1), and B3 (n = 1)). All fractures except one were treated closed. Open reduction was done in one patient who had four weeks old fracture. This was supplemented with cancellous bone graft, harvested from iliac crest.

The patient was positioned supine on a radiolucent operating table. The tibial medullary canal was accessed through an incision just medial to the patellar tendon and the nail introduced along the long axis of the tibia after making the entry point by bone awl.

Reaming was done in all cases over a guide wire, which was passed across the fracture site after obtaining a satisfactory reduction. Wherever the expandable nail of 8.5 mm diameter (collapsed state) was used, reaming was done up to 10/10.5 mm, whereas in cases where 10 mm nail was used, reaming was done up to 11/11.5 mm. This ensured smooth nail insertion. After reaming, the guide wire was removed gently, caution being exercised to maintain the reduction. Once the nail was satisfactorily placed in the medullary canal, it was inflated by pumping in normal saline up to a maximum pressure of 70 atmospheres. Satisfactory expansion of the nail was confirmed under image intensifier.^2^,^5^,^9^ After removal of the insertion device, the nail cap was put in the proximal end of the nail.^10^ In all the patients where bone graft was used, temporary immobilization with Thomas-Knee splint in femur fractures and long leg plaster slab applied in tibial fractures until the earliest radiological evidence of callus formation was observed.

Postoperatively, patellar tracking (horizontal and vertical manual patellar movement) and static quadriceps exercises were instituted as soon as feasible, titrated to patient's comfort. Partial weight-bearing was allowed, on regaining limb control, with the help of axillary crutches or walker. It promoted axial collapse at fracture site within physiological parameters. In the follow up, load bearing was gradually increased, depending upon clinical and radiological progress of union. However, in patients in whom cancellous bone grafting was done, load bearing was advised only when evidence of callus formation was visible, approximately in 4–6 weeks time. Initially, the patients were followed up at 2, 6, and 12 weeks, while subsequent follow-up was advised in every 3 months, for at least one year. Healing was considered complete when both clinical and radiological criteria of union were met. Clinical criteria for union were absence of localised tenderness at the fracture site and absence of pain on walking. Radiographic criteria of union were based on continuity in at least three cortices in both AP and lateral views.^5^ Specific note was taken for evidence of complications such as infection, malunion, delayed union, nonunion, shortening, and implant failure. As per the criteria used by Smith *et al*, malunion was defined as >5 degrees of angulation in any plane; nonunion was defined as absence of healing at 9 months or no sign of progression of healing at 3 months, and delayed union was defined as lack of evidence of radiographic healing or progression at 6 months.^11^

## RESULT

**Figure 4:**
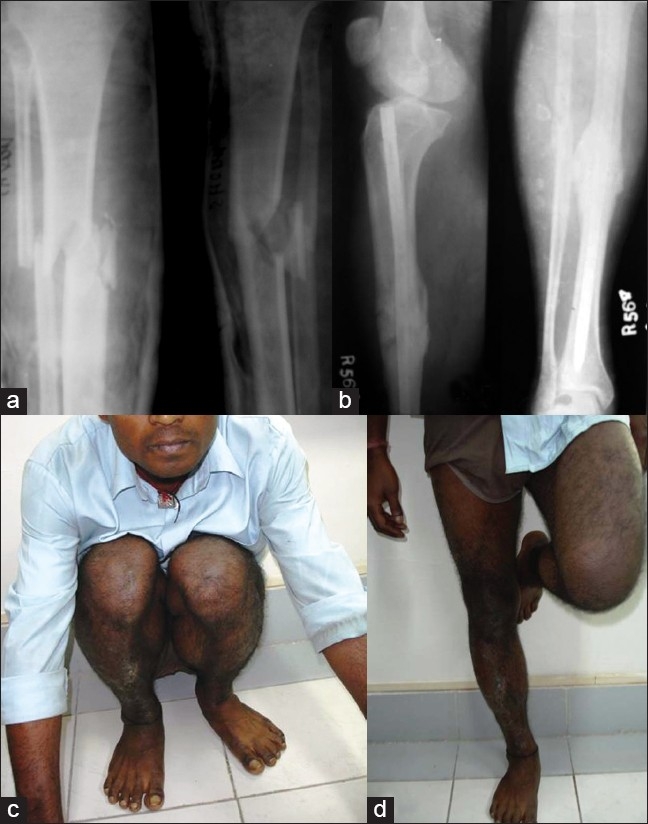
(a) Pre-operative X-rays showing diaphyseal fracture of middle third of tibia and fibula. (b) Follow up X-rays showing expandable nail in place and fracture consolidation. (c,d) Clinical function at final follow up

In our series, the modes of injury were road traffic accidents (n = 21), fall from height (n = 8), simple fall (n = 2), and pathological fracture (1). The median time of surgery since injury was 7.3 days (range, 1–32 days) for femoral fractures and 5.8 days (range, 6 h – 28 days) for tibial fractures. The average operative time was 90 min (range, 55–125 min) for femoral fractures and 53 min for tibial fractures (range, 25–115 min). The average radiation exposure time was 84 seconds (range, 54-132 seconds) for femoral fractures and 54 seconds (range, 36–78 seconds) for tibial fractures. Average blood loss (intraoperatively + drain collection) was 90 ml (55–135 ml) when closed method was used, and 270 ml (min 235ml to max 330 ml) when open reduction was done. We did not observe any neurovascular injuries, intra-operative extension of fracture, iatrogenic comminution, compartment syndrome, or fat embolism in our series. One patient of tibial fracture developed early surgical site infection. It was successfully managed by debridement, intravenous antibiotics, and dressing. This particular fracture went into delayed union where healing was achieved at 9 months [Figure 4a,b,c,d]. Immediate postoperative radiographs showed good alignment in all cases. One patient sustained a repeat trauma where the femoral nail was bent. The patient was managed conservatively and the fracture ultimately malunited at 10° of angulation and shortening of 1½ cm, as the patient refused further surgery. Two patients of femoral fracture went into delayed union, which responded to autologous bone marrow injection. The fractures united in 9 and 10½ months, respectively. We did not observe any nonunion or rotational malalignment.

The average time for clinical healing was 11.5 weeks (range, 8–28 weeks) in femoral fractures and 11 weeks (range, 8–25 weeks) in tibial fractures. The average time of radiological healing was 13.5 weeks (range, 10–44 weeks) in femoral fractures and 12.5 weeks (range, 9 –38weeks) in tibial fractures. Ultimately the healing was achieved in all the cases. The average time of healing for femoral fractures was 5.1 months (range, 4 –10½ months) and for tibial fractures was 4.8 months (range, 3–9 months) [Tables 2 and 3].

**Table 2:** Clinical data of patients with femur fractures

Age/sex	AO type	Mode of injury	Time since injury (days)	Duration of surgery (min)	Blood loss	Time of radiation exposure (s)	Healing time (months)	Complications
25/M	A3	RTA	5	65	60	54	4	Nil
25/M	A3	RTA	5	55	70	60	9	Delayed union
24/M	B1	RTA	4	65	135	132	4.1	Nil
32/F	A2	RTA	7	105	100	114	4	Nil
41/F	B3	FH	9	125	120	120	10.5	Delayed union
48/M	A1	FH	1	130	105	84	4	Nil
18/M	A3	RTA	1	70	90	78	5	Bent nail with malunion
28/F	A2	RTA	1	70	100	84	4.1	Nil
43/M	B2	FH	3	105 (open)	235	84	4	Nil
45/F	A3	RTA	5	100	95	66	4.2	Nil
23/M	A3	RTA	6	105	70	60	4.3	Nil
51/M	A2	RTA	7	110 (open)	75	84	5	Nil
22/F	A3	FH	8	95	85	90	5.2	Nil
62/F	A2	FS	11	70	90	96	5.3	Nil
42/M	B1	RTA	14	85	95	90	6	Nil
20/M	A3	P	1	80	235	84	4.3	Nil
28/M	A3	RTA	2	85	105	96	4	Nil
40/M	A1	RTA	32	85 (open)	330	66	5	Nil
29/M	B2	FH	3	100	110	126	5	Nil
18/M	A3	RTA	28	110 (open)	285	60	6	Nil
42/M	A1	RTA	3	90	105	66	4	Nil
22/F	A3	RTA	4	70	120	54	4	Nil

RTA= road traffic accident, FH= fall from height, FS= simple fall, P= pathological, M= male, F= female

**Table 3:** Clinical data of patients with tibia fractures

Age/Sex	AO Type	Mode of Injury	Time since injury (days)	Duration of surgery (min)	Blood loss	Time of radiation exposure (s)	Healing time (months)	Complications
24M	A3	RTA	28	60 (open)	265	54	9	Infection and DU
18M	A1	RTA	1	45	60	72	3.5	Nil
26M	A2	RTA	2	55	65	36	5	Nil
25M	B2	FH	1	40	80	66	4	Nil
27F	B1	RTA	2	65	100	72	5	Nil
30M	A2	RTA	4	35	115	36	4.5	Nil
45M	A1	RTA	7	50	65	48	3	Nil
55F	A3	FH	10	25	80	42	6	Nil
20M	B3	RTA	1	115	70	78	4.5	Nil
22M	A2	RTA	2	40	55	36	3.5	Nil

RTA= road traffic accident, FH= fall from height; FS= simple fall, P= pathological, M= male, F= female, DU=delayed union

Until the last follow-up, no nail had been removed. The minimum period of follow-up was 12 months and the maximum was 43 months. None of the patients was lost to follow-up.

## DISCUSSION

Diaphyseal fractures of femur and the tibia are common after high-energy trauma, particularly following road traffic accidents. In our series, majority of patients (21 out of 32) had road traffic accidents. Interlocked intramedullary nail is the universally accepted fixation device for closed diaphyseal fractures of long bones. It is a load-sharing implant and consistent satisfactory results have been reported with their use.^3^,^9^

These conventional nails require interlocking screws at both ends for axial and rotational stability. This increases the intra-operative radiation exposure as well as the operative time. The expandable nail eliminates the need for insertion of inter locking screws and hence less radiation exposure. The nail takes the exact shape of the medullary canal, and it remains in close contact with the entire endosteal surface (after expansion) compared with the interlocking nail which has only three points of fixation.^3^ The apex of the nail must exceed the fracture line by at least 5 cm to ensure good fixation.^2^ Since the stability of expandable nail is dependent upon close fit along the entire medullary canal, this type of nail is not suitable for fractures through those areas of diaphyses where the medullary canal is very wide. Consequently, this nail is not recommended in fractures of distal third of femur and distal most part of tibia where the nail can neither have a close fit with the endosteal surface at fracture site nor have a good hold to the wide distal fragment.

The small cross section of the collapsed nail allows its easy insertion.^2^ In femoral fractures, the trochanteric insertion through a relatively small entry portal eliminates the risk of iatrogenic fracture of femoral neck as sometimes encountered in cases of conventional nailing through the pyriformis fossa. This was made further easier by preoperative reaming in all our cases. The reaming done up to 2 mm more than the diameter of the nail, successfully avoided the complication of fracture propagation.^2^,^6^ We did not encounter any case of fat embolism, which is a reported complication of reaming.^3^ On the other hand, it would have definitely provided internal bone graft. The large spaces between the bars also allow rapid revascularization.^4^

Since the nail is not cannulated, the guide wire has to be removed before the insertion of the nail. So the surgical team has to be very cautious not to loose the fracture reduction after removal of the guide wire.^2^

Since the interlocking screws are not required to achieve stability with expandable nail, radiation exposure to the surgical team and the patient is minimized. The average radiation exposure in our series was 84 seconds for femoral fractures and 54 seconds for tibial fractures, which is comparable to the experiences of other workers.^7^,^11^–^13^ Smith *et al.*^11^ observed the average fluoroscopy time to be 110 seconds (range, 32–240 seconds). The total duration of radiation exposure is much less compared with the reported radiation exposure with the use of conventional interlocking nail.^1^,^14^

Other complications inherent to the use of locking screws are also eliminated namely, nerve (between 3.7% and 30%)^5^ and vessel injuries, increased stress at the site of screw insertion, compromised strength of nail because of screw holes, need for dynamization and hence second surgery, besides additional surgical scars, which may be of concern to patients, especially to the fairer sex.^3^–^5^,^7^ Since the need for screw insertion is eliminated, the operative time is also reduced. In our series, we spent an average of 90 min (range, 55–125 min) for femoral fractures and 55 min (range, 35–105 min) for tibial fractures. Smith *et al.*^11^ observed an average operative time of 44 min for femoral fixation and 41 min for tibial fractures. Lepore *et al.*^3^ observed average operative time of 2.4 ±0.6 h for femoral fixation, whereas Fortis *et al.*^15^ observed an average time of 40 ± 12.17 min for tibial fixation. In comparison, femoral fractures managed with conventional interlocking nails have been found to have an average operative time of 3.1 ± 0.5 h.^3^

In our study, there were few complications. There was no case of nonunion, but we had two cases of delayed union (lack of evidence of radiological healing or progression at 6 months)^11^ in 2 femoral fractures. In both the cases, union could be achieved by injection of autologous bone marrow, and there was no need for open bone grafting. One out of 32 cases with type B2 fracture of tibia had early surgical site infection [Figure 4]. The infection could be overcome by surgical debridement, intravenous antibiotic and dressing, but the fracture took 9 months to unite. One patient of femoral fracture slipped at home 6 weeks after the surgery and developed bending of the nail with anterolateral angulation at the fracture site, although X-ray showed early callus formation. The patient refused for second surgery. The fracture ultimately healed with an anterolateral angulation of 10° and 1½ cm shortening. Pascerella *et al.* have also reported one case of re-fracture of the femur with flexion of the expandable nail, which they attributed to be also due to second trauma.^2^ No other patient had any significant shortening (1 cm or more).

As the procedure is less time consuming and the implant is a self-locking device, it can be used in multiple fractures and polytrauma patients, where the fractures demand rapid fixation.^16^

## CONCLUSION

We are inclined to conclude that expandable self-locking nail is a simple and effective implant for the fixation of simple diaphyseal fractures of femur and tibia. It has definite advantages of reduced operative time and less radiation exposure in comparison with reported series of conventional interlocking nails.
